# Early treatment of patient with Class III skeletal and dental patterns

**DOI:** 10.1590/2177-6709.20.6.097-109.bbo

**Published:** 2015

**Authors:** Marcos Alan Vieira Bittencourt

**Affiliations:** 1 Associate professor of Orthodontics, Universidade Federal da Bahia (UFBA), Salvador, Bahia, Brazil.

**Keywords:** Angle Class III malocclusion, Crossbite, Interceptive Orthodontics

## Abstract

Class III skeletal pattern is characterized by disharmony between maxillary and mandibular basal bones anteroposteriorly, and might or might not be associated with dental changes. In general, facial esthetics is hindered significantly, which most of times is the reason why patients or patient's guardians seek treatment. This case was presented to the Brazilian Board of Orthodontics and Dentofacial Orthopedics (BBO) in partial fulfillment of the requirements for Diplomate recertification and revalidation.

## Resumo

O padrão esquelético de Classe III é caracterizado pela desarmonia entre as bases ósseas maxilar e mandibular, no sentido anteroposterior, podendo ou não estar acompanhada por alterações dentárias. Em geral, o aspecto estético facial fica bastante comprometido, sendo justamente esse o fator que, na maioria das vezes, motiva o paciente ou seus responsáveis a procurar pelo tratamento. Este caso clínico foi apresentado à Diretoria do Board Brasileiro de Ortodontia e Ortopedia Facial (BBO) como parte dos requisitos para o processo de recertificação e revalidação do título de Diplomado pelo BBO.

## INTRODUCTION

The present study reports the case of a 6 year and 3-month-old patient, in good general health, who sought treatment accompanied by her parents. Her medical history was uneventful, and she presented with good oral hygiene, without caries or restorations. She avoided smiling and the chief complaint reported by her parents was the presence of anterior crossbite and protruded chin and lower lip, which hindered facial esthetics. In general, some of these factors are determined by a hereditary component; however, the etiology of the case seemed multifactorial, based on patient's parents' facial esthetics and absence of this type of facial features in other members of their family.

## DIAGNOSIS

**Figure 1:**
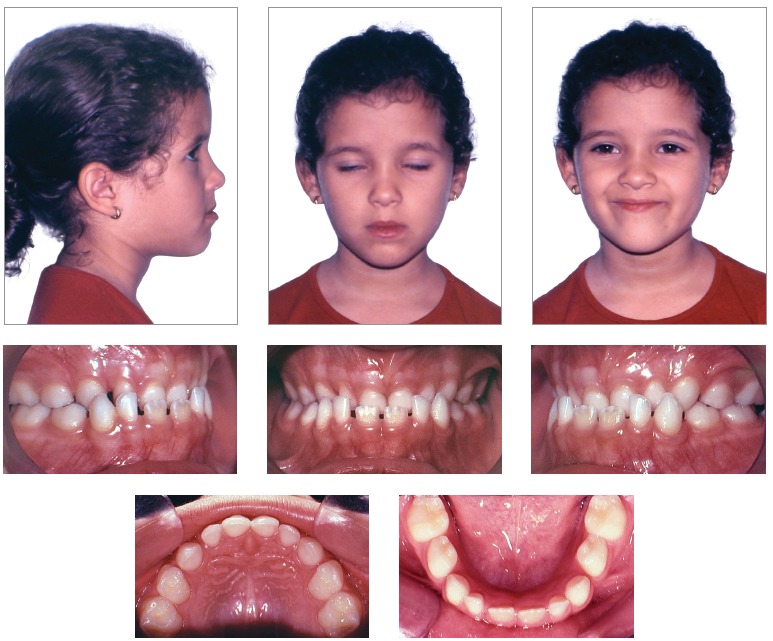
- Initial facial and intraoral photographs.

As shown in Figure 1, the patient presented with passive lip seal, slight deviation of the mandible to the right, which resulted in a slightly asymmetrical face, and deficient midface anteroposteriorly. She avoided smiling and showing her incisors teeth, which hindered assessment of spontaneous smile and the amount of tooth exposure at smile. The patient had a concave profile, with upper lip retrusion and lower lip protrusion, a proportional nose and increased nasolabial and labiomental angles.

**Figure 2:**
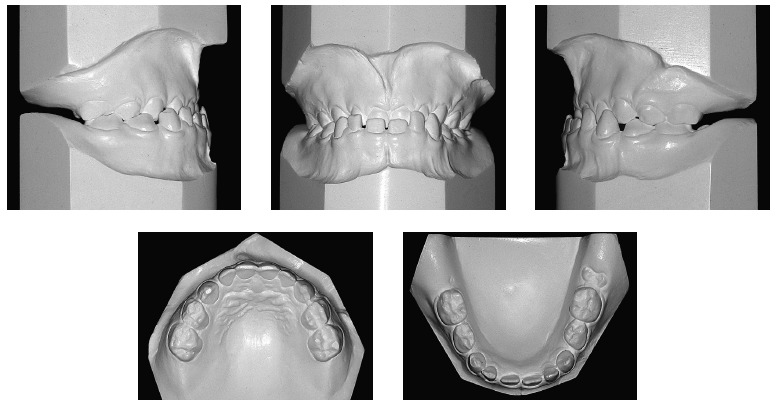
- Initial casts.

From a dental perspective (Figs 1, 2), she was in a rather incipient phase of mixed dentition, with early eruption of teeth #36, 31 and 41. She had Angle Class III malocclusion, with the terminal plane of deciduous mandibular second molars in mesial step. Also, the patient presented with Baume type I arches,^1^ with primate spaces in both upper and lower arches, 4-mm overbite and 1-mm overjet, maxillary dental midline coinciding with the median sagittal plane and 1-mm mandibular dental midline deviation to the right. In addition to anterior crossbite affecting the entire anterior region, she also had end-to-end bite of teeth #54 and 55 in the transverse plane, caused by mild maxillary atresia pronounced in the anterior region.

**Figure 3:**
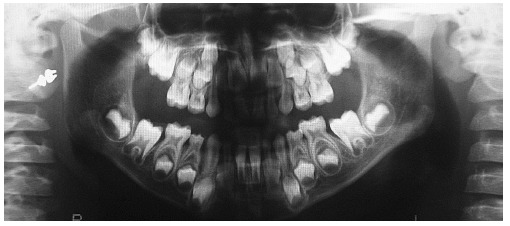
- Initial panoramic radiograph.

**Figure 4:**
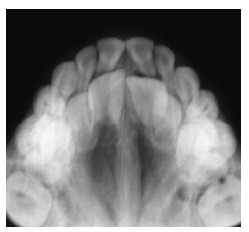
- Initial occlusal radiograph of the maxilla.

Analysis in panoramic radiograph (Fig 3) revealed normality of all deciduous and permanent teeth at different stages of root formation. Permanent maxillary and mandibular second molars were at an early stage of development, Nolla's stage 4,^2^ rendering it impossible to identify the onset of third molars formation. Additionally, occlusal radiograph of the maxilla (Fig 4) revealed that the region of the midpalatal suture was within normality standards.

**Figure 5:**
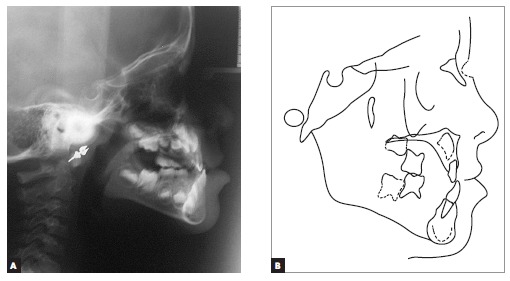
- Initial lateral cephalogram (A) and cephalometric tracing (B).

Lateral cephalograms and cephalometric tracings (Fig 5 and Table 1) revealed a Class III skeletal pattern (ANB = 0° and Wits = -7 mm), with retrusion of the maxilla in relation to the base of the skull, and protrusion of the mandible (Facial Angle = 91°). Slightly increased vertical growth pattern in the posterior region of the mandible, with a decreased mandibular plane (FMA = 21° and Y-axis = 54°), was also identified. As previously clinically observed, the patient had a concave profile, with upper lip retrusion (Upper Lip - S-line = -1 mm) and significant lower lip protrusion (Lower Lip - S-line = 4 mm). Mandibular incisors were retruded and in slightly upright position (1-NB = 20° and 1 mm).

**Table 1:** - Initial (A) and final (B) cephalometric values.

	**Measurements**		**Normal**	**A**	**A** ^1^	**A** ^2^	**B**	**Dif. A/B**
Skeletal pattern	SNA	(Steiner)	82°	81°	84°	81°	82°	1
	SNB	(Steiner)	80°	81°	79°	79°	80°	1
	ANB	(Steiner)	2°	0°	5°	2°	2°	2
	Wits	(Jacobson)	♀ 0 ±2 mm ♂ 1 ±2 mm	-7 mm	-1 mm	-2 mm	-2 mm	5
	Angle of convexity	(Downs)	0°	1°	9°	2°	2°	1
	Y-axis	(Downs)	59°	54°	55°	57°	60°	6
	Facial angle	(Downs)	87°	91°	90°	91°	88°	3
	SN-GoGn	(Steiner)	32°	31°	33°	31°	31°	0
	FMA	(Tweed)	25°	21°	23°	22°	24°	3
Dental pattern	IMPA	(Tweed)	90°	88°	87°	85°	89°	1
	1.NA (degrees)	(Steiner)	22°	-	12°	19°	19°	-
	1-NA (mm)	(Steiner)	4 mm	-	0 mm	4 mm	5 mm	-
	1.NB (degrees)	(Steiner)	25°	20°	19°	15°	20°	0
	1-NB (mm)	(Steiner)	4 mm	1 mm	4 mm	1 mm	5 mm	4
	- Interincisal angle	(Downs)	130°	-	142°	145°	140°	-
	1-APo	(Ricketts)	1 mm	2 mm	0 mm	1 mm	5 mm	3
Profile	Upper lip - S-line	(Steiner)	0 mm	-1 mm	1 mm	-1 mm	-2 mm	1
	Lower lip - S-line	(Steiner)	0 mm	4 mm	3 mm	2 mm	1 mm	3

As regards function, she presented with altered tongue positioning during speech and swallowing. With a view to analyzing whether anterior crossbite hindered function, patient's closure pattern was assessed, revealing no shift. In other words, centric relation position was very close to intercuspal contact position.

**Figure 6:**
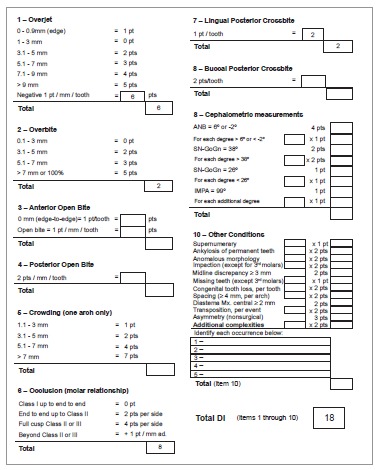
- Calculation of the Discrepancy Index (DI).

Discrepancy index (DI) was calculated and scored 18 points (Fig 6).

## TREATMENT PLAN

Taking facial esthetics and disharmony between the maxilla and mandible anteroposteriorly into account, orthodontic treatment was planned to be conducted in two phases. In the first phase, the goal was to achieve a balanced profile and restore lip posture as well as the skeletal relationship between the maxilla and the mandible. To this end, reverse maxillary protraction was planned to be carried out by means of Petit face mask.^3^ In order to render anterior repositioning of the maxilla easier, by means of weakening the sutures that hold the other facial bones together, in addition to enhancing its transverse dimension, palatal expansion^4^would be performed by the modified Haas expansion appliance. As a result, the anteroposterior relationship between the maxilla and the mandible would also be improved and anterior crossbite corrected.

Since there was positive discrepancy in the mandible, with potential presence of Lee Way Space^5^ by the end of the mixed dentition, treatment plan included using the remaining space for retrusion of mandibular teeth. Thus, before the second phase of treatment, a lingual bar would be installed immediately before exfoliation of deciduous mandibular second molars, thereby keeping permanent first molars in place and preserving the Lee Way Space.

In the second phase of treatment, conventional fixed orthodontic appliance would be installed in both upper and lower arches with a view to distalizing mandibular premolars and canines, in addition to retracting mandibular incisors by means of taking advantage of the space preserved as a result of using the lingual bar, thus improving the relationship between the maxilla and mandible in the transverse and anteroposterior planes. At this phase, it would also be important to improve Class I molar and canine relationship, adjust lateral protrusive guidance, and align and level all maxillary and mandibular teeth.

After conventional treatment completion, a wraparound removable appliance was planned to be used in the maxilla and a 0.028-in stainless steel intercanine bar to be bonded to mandibular teeth, both for retention. Special attention would also be given to the process of formation and development of third molars.

Based on the need for patient's compliance with the face mask and the uncertainty about achieving a harmonious relationship between maxillary and mandibular basal bones anteroposteriorly, an alternative treatment plan by the end of growth would be to wait for facial growth to cease and dentition as well as occlusion to fully develop before conventional orthodontic-surgical treatment was conducted. This treatment modality was rejected by the patient's parents who understood the biological limitations involved and undertook the responsibility of making an effort with a view to gaining patient' compliance.

## TREATMENT PROGRESS - FIRST PHASE

Orthodontic treatment began as planned, with the modified Haas appliance installed and supported by bands adapted to deciduous second molars and bonded to deciduous first molars and canines. Moreover, 0.032-in stainless steel wires were adapted and welded to the orthodontic bands buccaly. They were also adapted and welded to the buccal surface of the aforementioned teeth, with hooks placed mesial to canines and used as support for maxillary protraction. The patient was advised to perform 1/4 of a turn every 12 hours for 15 days. After this period, the expansion appliance was made stable and the patient began to use the Petit face mask, with 400-g force application on each side, 18 hours a day. It is worth noting that the face mask was adapted so as to allow the elastics to form a 30° angle with the occlusal plane, thereby moving the maxilla forward without undesired counterclockwise rotation.^6^

The patient was followed up every two months. Six months after treatment onset, the use of the face mask decreased to eight hours a day (at night); six months after that, the use of the face mask ceased completely and the expansion appliance was removed. Improvements resulting from the first phase of treatment can be seen inFigures 7 to 10.

**Figure 7:**
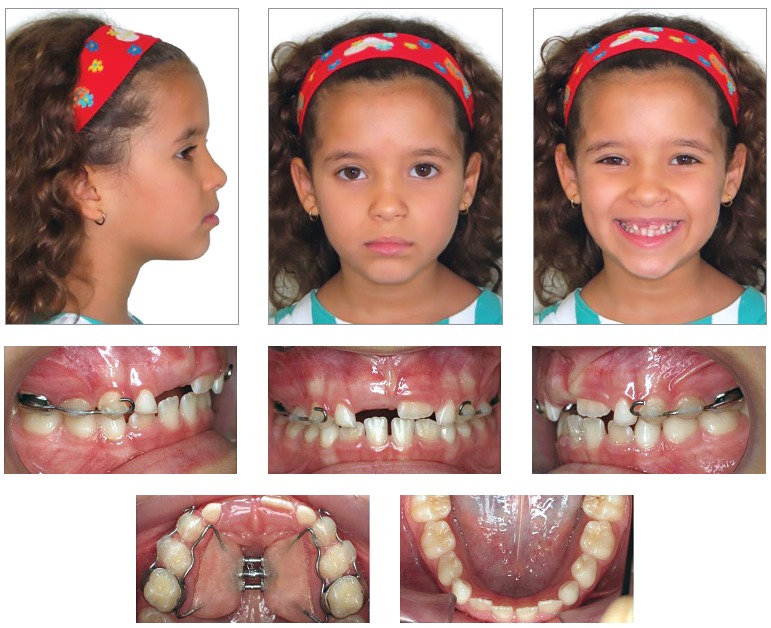
- Facial and intraoral photographs at first treatment phase completion, immediately before removal of the Haas appliance.

**Figure 8:**
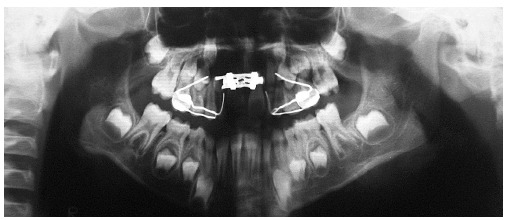
- Panoramic radiograph at first treatment phase completion, immediately before removal of the Haas appliance.

**Figure 9:**
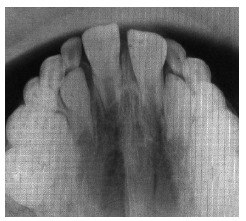
- Occlusal radiograph of the maxilla at first treatment phase completion, immediately before removal of the Haas appliance.

**Figure 10:**
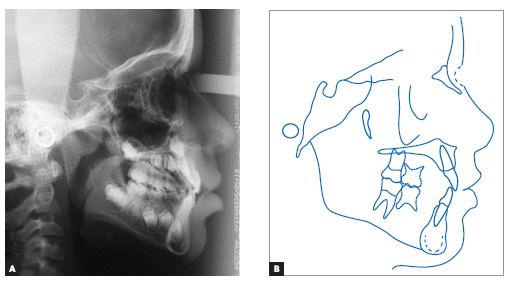
- Initial lateral cephalogram (A) and cephalometric tracing (B) at first treatment phase completion, immediately before removal of the Haas appliance.

After three years and six months of following up patient's occlusion development, with appointments arranged every six months, the lingual bar was installed, as planned. At this point, the patient was 11 years and one month old. Clinically, she presented with a straight profile, passive lip seal, proportional facial thirds and a pleasant smile. From a dental perspective, she was near the end of mixed dentition, at the second transitional period; however, with increased exfoliation in the mandible where second molars were the only deciduous teeth left. As for the maxilla, in which the exfoliation process was delayed, deciduous canines, first and second molars were present.

**Figure 11:**
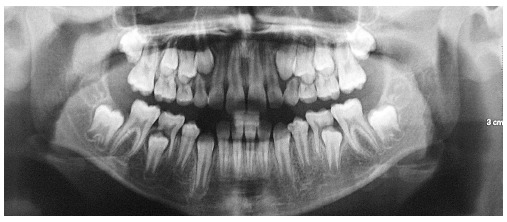
- Panoramic radiograph at 11 years and one month old.

Panoramic radiograph (Fig 11) revealed teeth #35 and 45 slightly distally proclined, thereby inciting distal root resorption of teeth #75 and 85, respectively. Mesial roots remained practically completely intact, with root resorption beginning near the cervical third.

In this scenario, it was decided to install the lingual bar made of 0.032-in stainless steel wire welded to orthodontic bands adapted to teeth #36 and 46. Subsequently, the patient was referred for extraction of teeth #75 and 85 with a view to physiologically improving the eruption axis of teeth #35 and 45. As the goal was to preserve Lee Way Space in the mandible, so as to take advantage of the remaining space to move mandibular teeth posteriorly while minimizing Class III skeletal pattern expression, the patient was followed up until exfoliation of all deciduous teeth and eruption of their permanent successors.

## TREATMENT PROGRESS - SECOND PHASE

**Figure 12:**
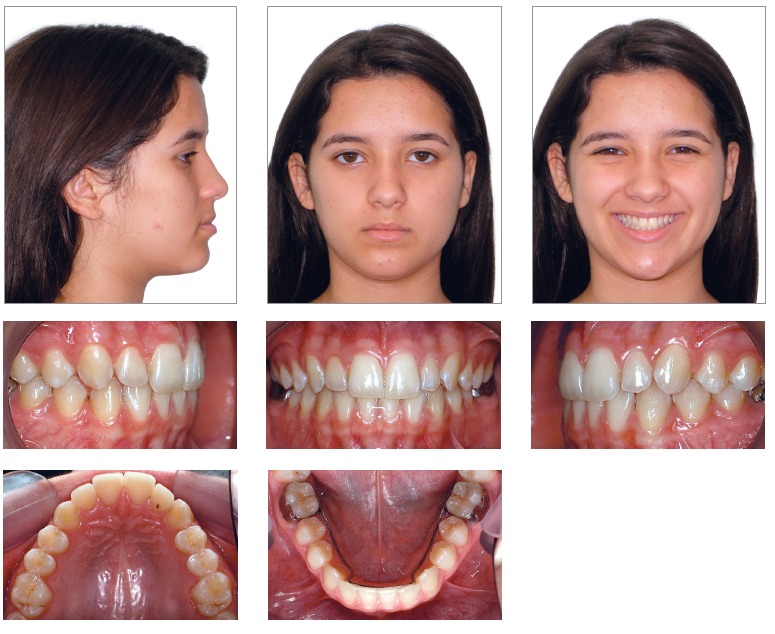
- Facial and intraoral photographs at second phase of treatment onset.

**Figure 13:**
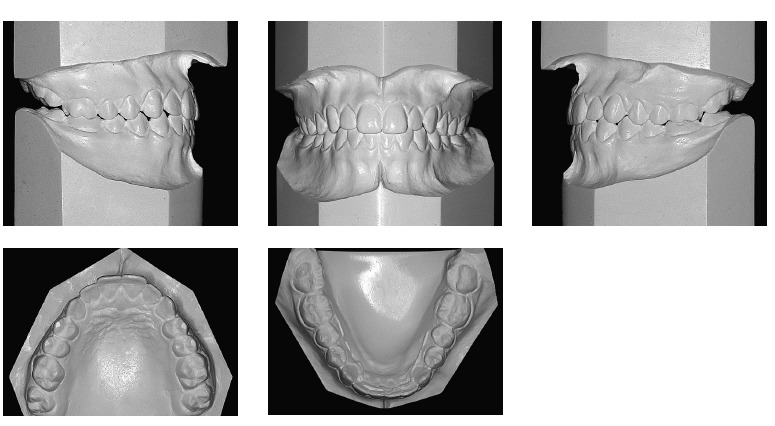
- Casts at second phase of treatment onset.

**Figure 14:**
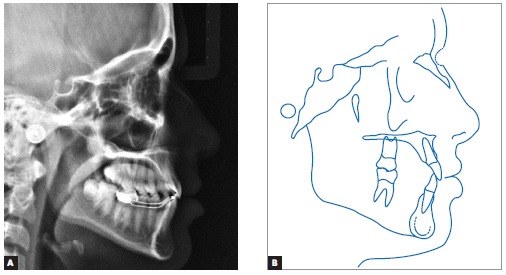
- Lateral cephalogram (A) and cephalometric tracing (B) at second phase of treatment onset.

After three years and seven months following up patient's occlusion development, with appointments arranged every six months, the second phase of treatment began. At this point, the patient was 14 years and eight months old. Clinically, as shown in Figure 12, she remained with a straight profile, satisfactory passive lip seal, proportional facial thirds and an esthetically pleasant smile. From a dental perspective (Figs 12 to 14), she had all permanent teeth erupted, including second molars, except for third molars.

Analysis of the stages of maturation of cervical vertebrae C2, C3 and C4 (Fig 14) revealed that the indicators of skeletal maturity signaled to the end of the third growth spurt.^7^

In view of the above, the second phase of treatment began with conventional orthodontic fixed appliance bonded to all teeth in both upper and lower arches, with metal brackets, Edgewise standard technique, without torques or angulations, and with 0.022 x 0.028-in slots.

Subsequently, alignment and leveling 0.014 to 0.020-in stainless steel round archwires were placed in the maxilla and mandible. Additionally, 0.018 x 0.025-in rectangular archwires were installed in the mandible. A tie together ligature was made with the 0.020-in mandibular archwire between the first and second molars on both sides. An elastomeric chain was placed to slightly move second premolars distally and correct the mild torsiversion presented by tooth #35. Subsequently, these teeth were also included as anchorage units, and elastomeric chains were individually used in order to slightly move first premolars and canines distally. Once the 0.018 x 0.025-in stainless steel archwire had been placed, an elastic tie back bend was crimped at the lower arch. Meanwhile, Class III intermaxillary mechanics was applied, as anchorage reinforcement, with a view to closing the remaining spaces mesial to canines by means of slight retraction of incisors.

At the following phase and after all spaces had been closed, 0.019 x 0.025-in stainless steel rectangular archwires, with first- and third-order bends placed as necessary, were placed in both upper and lower arches for treatment finishing.

Once all treatment goals had been achieved, the orthodontic fixed appliance was removed and the retention phase started. A wraparound removable appliance was placed in the upper arch and a fixed intercanine bar, manufactured with 0.028-in stainless steel round wire, was bonded to the lower arch. The patient was advised to use the retainer in the upper arch 24 hours a day for the first six months, 18 hours a day for the following six months, 12 hours a day for the following six months after that, and then at night, only.

## RESULTS

**Figure 15:**
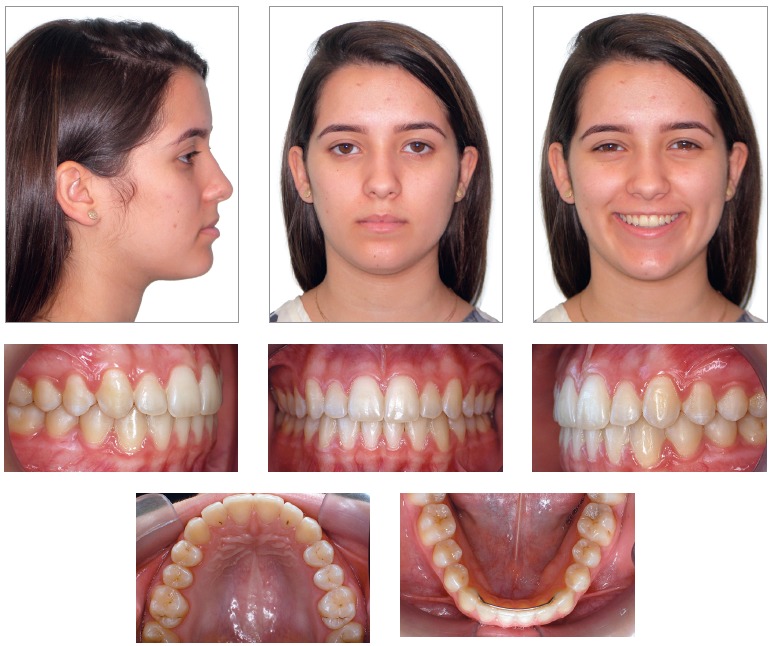
- Final facial and intraoral photographs.

**Figure 16:**
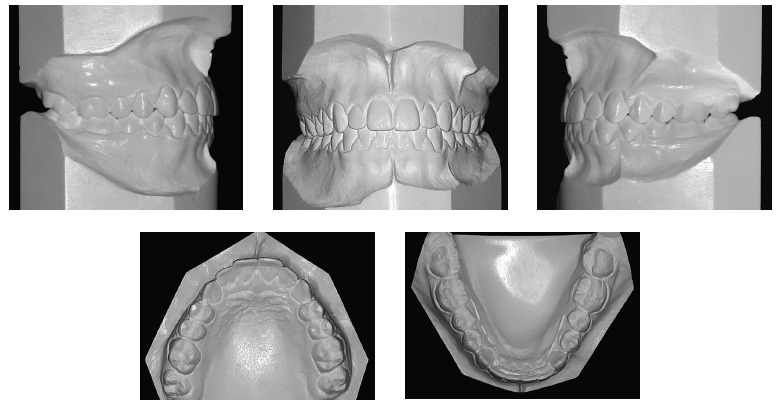
- Final casts.

**Figure 17:**
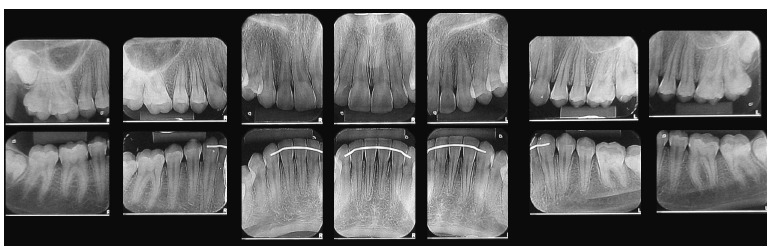
- Final periapical radiographs.

**Figure 18:**
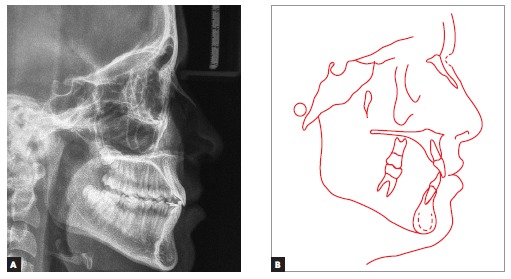
- Final lateral cephalogram (A) and cephalometric tracing (B).

Patient's final records (Figs 15 to 18) assessment revealed that all treatment objectives were achieved. As regards patient's face, a comparison between final and initial records taken at orthodontic treatment onset revealed significant profile improvement, significant movement of the upper lip forward and adjustment of the lower lip, thereby resulting in an esthetic profile, with better nasolabial and labiomental angles contour. Frontal analysis revealed a balanced face, with the mandible symmetrically positioned in relation to the median sagittal plane. The patient also presented with good passive lip seal, proportional facial thirds and an esthetically pleasant smile, which resulted in excellent smile arc contour and adequate buccal corridor dimensions.^8^

Tooth analysis revealed adequate alignment and leveling and Class I molar and canine relationship achieved on both sides. Transverse deficiency, anterior crossbite and lower midline deviation were corrected. There was slight intentional increase in maxillary intercanine width from 32 mm to 34 mm. Mandibular intercanine width remained at 26 mm. Functional harmony was excellent for occlusion in protrusive excursion and right as well as left lateral guidance, with centric relation coinciding with maximal intercuspation position. It is worth noting that, as shown by periapical radiographs taken at treatment completion (Fig 17), changes were achieved without radiographically noticeable apical root remodeling.

As planned, cephalometric examination revealed a number of skeletal changes. A-Point moved slightly forward while B-point moved backwards, thereby resulting in skeletal pattern correction, with Wits value from -7 mm to -2 mm, which is within normality standards for female patients.^9^ These changes also led to facial angle improvement from 91° to 88°, a value near normality standards. In addition, there was slight opening up of the mandibular plane angle (Y-axis from 54° to 60° and FMA from 21° to 24°), which resulted in significant improvement in proportionality between facial thirds in the vertical plane. These skeletal changes were also responsible for improvements in the relationship between upper and lower lips, with the lower lip positioned from 4 mm to 1 mm in relation to the S-line,^10^ which resulted in an esthetically pleasant profile. These data are shown in Figure 18 and Table 1.

**Figure 19:**
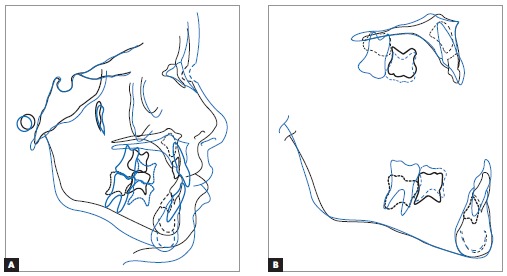
- Total (A) and partial (B) cephalometric superimpositions of initial (black) and at first treatment phase completion (blue) tracings.

Cephalometric superimposition revealed that, in the first phase of treatment (Fig 19), there was significant maxillary advancement forward, correcting overjet; and slight clockwise rotation of the mandible, opening up the mandibular plane angle and significantly improving patient's facial profile while also enhancing upper and lower lips position. Partial cephalometric superimposition revealed a decrease in maxillary length, with a reduction between incisors and deciduous second molars width due to anchorage loss of the latter. This was potentially followed by displacement of the maxilla forward and slight vertical growth of the alveolar process. Slight vertical growth of the alveolar process was also seen in the mandible, followed by slight mandibular growth.

**Figure 20:**
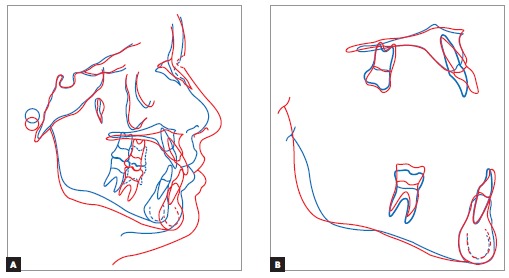
- Total (A) and partial (B) cephalometric superimpositions of at first treatment phase completion (blue) and final (red) tracings.

Between the first phase of treatment and treatment completion (Fig 20), there was harmonious growth of the entire facial complex forward and downward, with profile balance preserved. In the maxilla, there was a major improvement in incisors proclination; from significant upright position to a much favorable position. As for molars, there was slight anchorage loss, occupying the Lee Way Space, and seeking improvement in Class I molar relationship with the mandibular tooth. In the mandible, there was a slight improvement in incisors proclination. As predicted and desired, molars positioning remained unchanged, without anchorage loss; in addition, there was vertical growth of the alveolar process followed by mandibular growth.

## FINAL CONSIDERATIONS

As previously reported,^11^ Angle Class III malocclusion, characterized by anteroposterior dental discrepancy, is rendered more severe when in association with skeletal disharmony which might result from maxillary deficiency, mandibular excess or a combination of both. These changes hinder facial profile, often leading to psychosocial consequences.

The therapeutic possibilities available to correct the anomaly imply in a number a factors. For patients in the pre-pubertal growth spurt phase, an early approach is recommended; for instance, the use of face mask for reverse maxillary protraction usually associated with palatal expansion.^12^With this approach, as performed in the case reported herein, a more forward positioning of the maxilla is expected, so as to enhance its relationship with the mandible, in addition to providing satisfactory occlusion and pleasant facial esthetics. Nevertheless, patient's guardians should be informed about a number of uncertainties involved in the potential outcomes to be achieved, especially regarding the direction of patient's facial growth^13^ and the degree of patient's compliance. Thus, in spite of agreeing with the role heredity plays in the etiology of Class III skeletal malocclusion, several authors believe it is possible not only to change growth pattern and direction by means of a non-surgical approach, but also to minimize or even treat the malocclusion successfully.^14^^,^ 15 Younger individuals tend to yield more favorable results, in which case the ideal age ranges between 4 and 10 years old, although patients aged between 10 and 14 years old also yield positive outcomes.^16^^,^ 17 Deguchi et al^18^ assessed, during six months, variations in Wits value for children treated with the same treatment modality described herein. The authors found an increase of 2 mm. For the clinical case reported by the present study, there was a greater improvement: 5 mm of change in Wits value.

In the first phase of treatment, maxillary protraction by means of Petit face mask^3^ was conducted in combination with rapid maxillary expansion, with a view to enhancing its transverse dimension while weakening the sutures that hold the other facial bones together, thereby rendering maxillary displacement forward effective.^19^Importantly, this was the main procedure carried out for the present clinical case, which allowed patient's mid- and lower face to grow and develop accordingly, thus significantly minimizing the complexity of the following phase. Furthermore, the stability of procedures carried out during the first phase of treatment reveals that the biological limits were respected,^20^^,^ 21 which resulted in favorable functional as well as esthetic outcomes.

## References

[1] Baume LJ (1950). Physiological tooth migration and its significance for the development
of occlusion. I. The biogenetic course of the deciduous dentition. J Dent Res.

[2] Nolla CM (1960). The development of the permanent teeth. J Dent Child.

[3] Petit HP (1982). The prognathic syndrome: a complete treatment plan around the facial
mask. Rev Orthop Dento Faciale.

[4] McNamara JA (1987). An orthopedic approach to the treatment of Class III malocclusion in
young patients. J Clin Orthod.

[5] Nance HN (1947). The limitations of orthodontic treatment; mixed dentition diagnosis
and treatment. Am J Orthod.

[6] Ngan PW, Hagg U, Yiu C, Wei SH (1997). Treatment response and long-term dentofacial adaptations to maxillary
expansion and protraction. Semin Orthod.

[7] Baccetti T, Franchi L, McNamara JA (2002). An improved version of the cervical vertebral maturation (CVM) method
for the assessment of mandibular growth. Angle Orthod.

[8] Nascimento DC, Santos ER, Machado AWL, Bittencourt MAV (2012). Influence of buccal corridor dimension on smile
esthetics. Dental Press J Orthod.

[9] Jacobson A (1975). The "Wits" appraisal of jaw disharmony. Am J Orthod.

[10] Steiner CC (1953). Cephalometrics for you and me. Am J Orthod.

[11] Bittencourt MAV (2009). Má oclusão Classe III de Angle com discrepância ântero-posterior
acentuada. Rev Dental Press Ortod Ortop Facial.

[12] Ngan P, Hägg U, Yiu C, Merwin D, Wei SH (1996). Soft tissues and dentoskeletal profile changes associated with
maxillary expansion and protraction headgear treatment. Am J Orthod Dentofacial Orthop.

[13] Ngan P (2006). Early treatment of Class III malocclusion: is it worth the
burden?. Am J Orthod Dentofacial Orthop.

[14] Araújo EA, Araújo CV (2008). Abordagem clínica não-cirúrgica no tratamento da má oclusão de Classe
III. Rev Dental Press Ortod Ortop Facial.

[15] Westwood PV, McNamara JA, Baccetti T, Franchi L, Sarver DM (2003). Long-term effects of Class III treatment with rapid maxillary
expansion and facemask therapy followed by fixed appliances. Am J Orthod Dentofacial Orthop.

[16] Baccetti T, McGill JS, Franchi L, McNamara JA, Tollaro I (1998). Skeletal effects of early treatment of Class III malocclusion with
maxillary expansion and face-mask therapy. Am J Orthod Dentofacial Orthop.

[17] Brunetto AR (2009). Má oclusão de Classe I de Angle, com tendência à Classe III
esquelética, tratada com controle de crescimento. Rev Dental Press Ortod Ortop Facial.

[18] Deguchi T, Kanomi R, Ashizawa Y, Rosenstein SW (1999). Very early face mask therapy in Class III children. Angle Orthod.

[19] Silva OG, Magro AC, Capelozza L (1998). Early treatment of the Class III malocclusion with rapid maxillary
expansion and maxillary protraction. Am J Orthod Dentofacial Orthop.

[20] Cericato GO, Freitas PH, Bittencourt MAV, Paranhos LR (2015). Reliability, efficacy and reproducibility of the cervical vertebrae
maturation index (CVMI). Biosc J.

[21] Cericato GO, Bittencourt MA, Paranhos LR (2015). Validity of the assessment method of skeletal maturation by cervical
vertebrae: a systematic review and meta-analysis. Dentomaxillofac Radiol.

